# Nucleobase-modified nucleosides and nucleotides: Applications in biochemistry, synthetic biology, and drug discovery

**DOI:** 10.3389/fchem.2022.1051525

**Published:** 2022-11-30

**Authors:** Anthony Berdis

**Affiliations:** Department of Chemistry, Cleveland State University, Cleveland, OH, United States

**Keywords:** DNA polymerization, nucleoside analogs, hydrogen bonding, synthetic biology, chemotherapy

## Abstract

Abstract. DNA is often referred to as the “molecule of life” since it contains the genetic blueprint for all forms of life on this planet. The core building blocks composing DNA are deoxynucleotides. While the deoxyribose sugar and phosphate group are ubiquitous, it is the composition and spatial arrangement of the four natural nucleobases, adenine (A), cytosine (C), guanine (G), and thymine (T), that provide diversity in the coding information present in DNA. The ability of DNA to function as the genetic blueprint has historically been attributed to the formation of proper hydrogen bonding interactions made between complementary nucleobases. However, recent chemical and biochemical studies using nucleobase-modified nucleotides that contain “non-hydrogen bonding” functional groups have challenged many of the dogmatic views for the necessity of hydrogen-bonding interactions for DNA stability and function. Based on years of exciting research, this area has expanded tremendously and is thus too expansive to provide a comprehensive review on the topic. As such, this review article provides an opinion highlighting how nucleobase-modified nucleotides are being applied in diverse biomedical fields, focusing on three exciting areas of research. The first section addresses how these analogs are used as mechanistic probes for DNA polymerase activity and fidelity during replication. This section outlines the synthetic logic and medicinal chemistry approaches used to replace hydrogen-bonding functional groups to examine the contributions of shape/size, nucleobase hydrophobicity, and pi-electron interactions. The second section extends these mechanistic studies to provide insight into how nucleobase-modified nucleosides are used in synthetic biology. One example is through expansion of the genetic code in which changing the composition of DNA makes it possible to site-specifically incorporate unnatural amino acids bearing unique functional groups into enzymes and receptors. The final section describes results of pre-clinical studies using nucleobase-modified nucleosides as potential therapeutic agents against diseases such as cancer.

## Introduction

**FIGURE 1:**
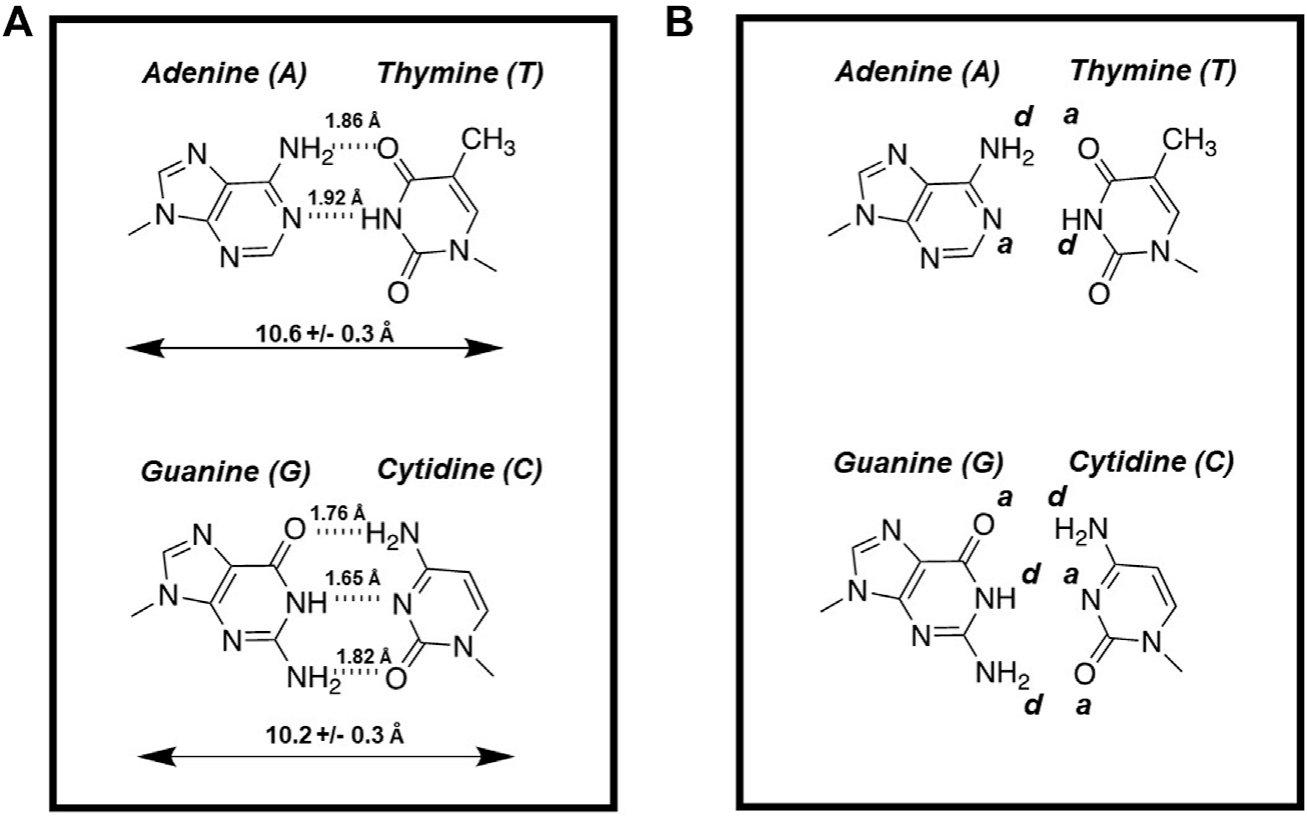
**(A)** Structures of Watson-crick base pairs are stabilized by hydrogen bonding interactions made between the complementary nucleobases. **(B)** Hydrogen bonding patterns for an A:T base pair which uses complementarity of d*a*(-) to a*d*a while a G:C base pair uses complementarity of a*d*d to d*a*a pairing.

DNA is a complex biopolymer that functions as the carrier of genetic information for most organisms on Earth. The double-stranded helical chain consists of nucleotides linked in a linear fashion that are complements to each other. A fundamental concept for understanding the structure of DNA as well as the chemical process of DNA replication involves specific hydrogen bonding interactions made between these complementary nucleobases (Figure 1A). These non-covalent forces not only help stabilize DNA but also contribute to base identification during the polymerization cycle. In this case, the mutual recognition of adenine (A) by thymine (T) and guanine (G) by cytosine (C) involves hydrogen bonding interactions between each partner. At the atomic level, NH and -NH_2_ groups serve as hydrogen bond donors (denoted as d) while oxygens present on C=O groups and unprotonated nitrogens are hydrogen bond acceptors (denoted as a). Figure 1B provides hydrogen bonding patterns for an A:T base pair which uses complementarity of d*a to a*d while a G:C base pair uses complementarity of a*d*d to d*a*a pairing. These base pairing patterns are commonly referred to as Watson-Crick base pairs (Engelhart and Hud, 2010).

While hydrogen bonding interactions are the most recognizable feature of DNA, other factors such as desolvation, pi-pi stacking, and geometrical constraints also contribute extensively to DNA structure and replication [reviewed in (Kool, 2001)]. Desolvation energy, the amount of energy required to remove water from a molecule, and hydrophobicity, the tendency of a molecule to repel water, are often used interchangeably. However, they have different biophysical meanings in the context of DNA polymerization. For example, the interior of the DNA helix is a hydrophobic environment since it is devoid of water, and this is essential in order for hydrogen-bonding interactions to occur between functional groups present on complementary nucleobases. However, during the polymerization process, both the templating nucleobase and incoming nucleotide containing hydrophilic functional groups must be desolvated in order to properly form hydrogen-bonds within this hydrophobic environment. Another important parameter is pi-pi stacking interactions between aromatic nucleobases that can define local structure and flexibility of DNA to influence the efficiency and fidelity of DNA synthesis. Finally, steric constraints imposed by the geometrical conformation of DNA and active site architecture of a DNA polymerase also influence nucleotide selection during DNA polymerization and exonuclease proofreading.

Perhaps the largest complication toward completely understanding the contributions of each of these features lies in the fact that hydrogen-bonding interactions, pi-electron density, solvation energies, and steric constraints are all interrelated. In essence, changing one feature can directly and indirectly influence the others. Indeed, modifying a hydrogen-bonding functional group on any nucleobase will alter its overall solvation energy as well as change its degree of aromaticity and overall shape. Having said that, it is remarkable that nucleobase-modified nucleotides possessing diverse chemical moieties have been developed and can function as efficient substrates for DNA polymerases.

This review article provides a scientific opinion of how nucleobase-modified nucleotides are being used in various biomedical fields. Please note that the analogs discussed here contain only modifications to the nucleobase and are thus different from analogs possessing alterations to either the sugar moiety or phosphate groups, respectively. These analogs have been referred to by many names including artificial nucleotides, unnatural nucleotides, and non-native nucleotides. Regardless of the difference in nomenclature, this article focuses on three major areas of contemporary research efforts to highlight their utility. The first section describes how these novel analogs are used as mechanistic probes for DNA polymerase activity and replicative fidelity. This section describes the chemical logic used to replace hydrogen-bonding functional groups as well as kinetic approaches to critically evaluate the contributions of shape and size, nucleobase hydrophobicity, and pi-electron stacking interactions to DNA polymerization. The second section describes how nucleobase-modified nucleosides are being developed to expand the genetic code, going from four base pairing combination (A:T, T:A, C:G, and G:C) to six possible combinations (A:T, T:A, C:G, G:C, X:Y, and Y:X). The ultimate goal here is to develop new base pairing combinations so that additional amino acids (beyond the twenty natural occurring amino acids) can be selectively incorporated into proteins during translation. The final section describes current efforts using nucleobase-modified nucleosides as potential therapeutic agents against hyperproliferative diseases such as cancer. Recent results highlight the ability to combine nucleobase-modified nucleosides with anti-cancer agents that damage DNA as a new therapeutic strategy to improve the overall efficacy and safety of existing chemotherapeutic agents.

## Nucleobase-modified nucleoside analogs as probes for DNA polymerase activity and fidelity

**FIGURE 2:**
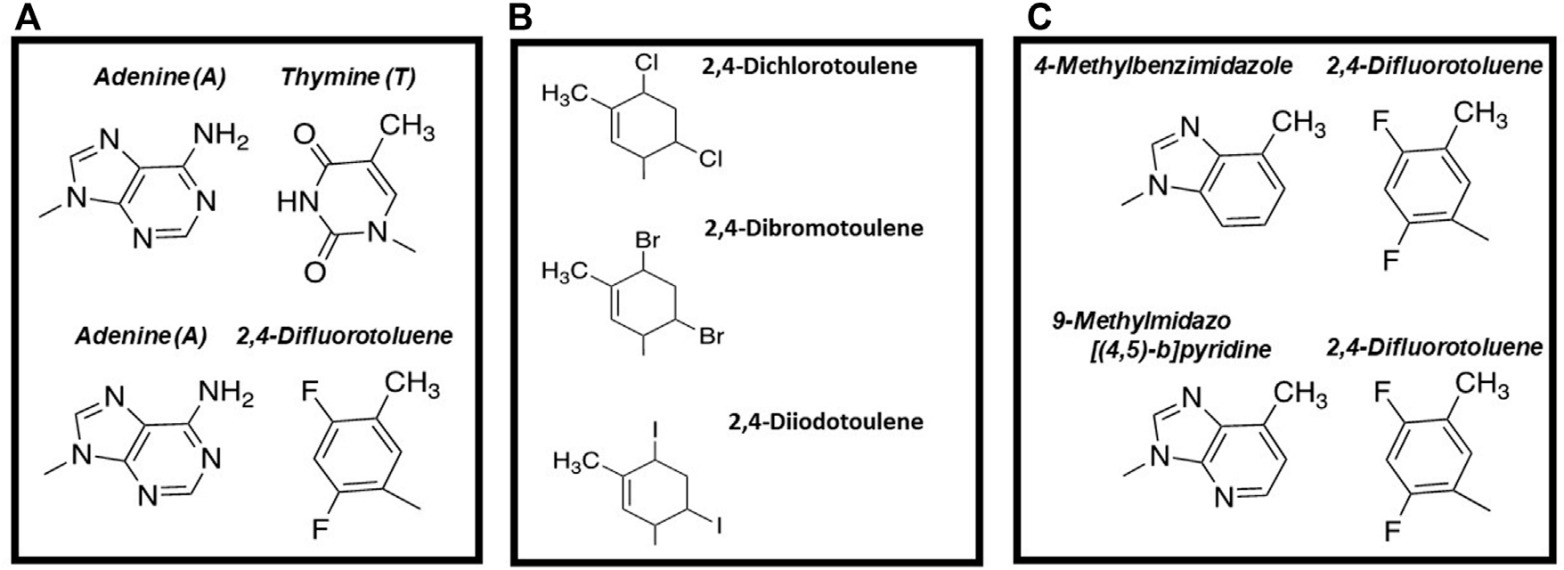
**(A)** Structural comparison of 2,4-difluorotoluene with thymine **(B)** Structures of thymidine analogs containing different halogens that vary in size **(C)** Comparison of mixed, nucleobase-modified base pairs including adenine: 2,4-difluorotoluene, 4-methylbenzimidazole:difluorotoluene, and 9-methylimidazo [ (Kool, 2002; Kool and Sintim, 2006)-b]pyridine: 2,4-difluorotoluene.

Hydrogen-Bonding Interactions Are Not Essential for DNA Polymerization. The most widely-cited study examining DNA polymerization in the absence of hydrogen-bonding interactions is work reported by Kool and colleagues demonstrating that 2,4-difluorotoluene triphosphate (Figure 2A), a non-hydrogen-bonding isostere of dTTP, is an effective DNA polymerase substrate (Moran et al., 1997). Despite lacking hydrogen bonding capabilities, this analog is incorporated opposite A with overall catalytic efficiency that is only 100-fold lower than that for incorporating dTTP opposite A. In addition, 2,4-difluorotoluene appears selective for insertion opposite A as the analog is poorly incorporated opposite G, C, and T. This is caused by reductions in the binding affinity for the nucleobase-modified nucleotide coupled with decreases in the rate of incorporation opposite these three natural nucleobases.

At the time of their publication, these results were groundbreaking as they demonstrated that canonical Watson-Crick hydrogen bonds are not required for DNA polymerization. This information was used to develop a new model for polymerization fidelity that is commonly referred to as “shape complementarity” model (Kool, 2002). This model invokes geometrical alignment of the incoming nucleobase with the template base as the predominant force for optimizing nucleotide selection (Kool, 2002; Kool and Sintim, 2006). Accordingly, the ability of a polymerase to distinguish between a correct *versus* incorrect dNTP depends upon the size and geometry of DNA polymerase’s active site in addition to interactions defined by size constraints imposed by the major and minor grooves of DNA (Kool, 2002).

While this model is elegantly simple, there are a few interesting and often overlooked findings from this initial study that diminish its overall validity. For example, it is striking that the catalytic efficiency for inserting 2,4-difluorotoluene triphosphate opposite 2,4-difluorotoluene as the templating nucleobase is higher than that measured for its incorporation opposite adenine (Moran et al., 1997). In this case, the maximal velocity (V_max_) for both incorporation events are essentially identical while the K_m_ value of 53 μM measured for 2,4-difluorotoluene triphosphate opposite itself is 2-fold lower than that of 95 μM measured opposite adenine. Thus, these data indicate that the nucleobase-modified nucleotide is more inclined to form a “self pair” as opposed to forming a “mixed” base pair that is similar in shape and size to a natural base pair. As described later, the “self-pairing” capability of 2,4-difluorotoluene is not unique as this phenomenon is observed with other nucleobase-modified nucleotides that lack conventional hydrogen bonding functional groups (*vide infra*).

Regardless, the Kool group continued to investigate the importance of steric effects by introducing different halides at the 2- and 4-position of toluene (Figure 2B). The strategy was to systematically increase the size of the base pair in small increments of 0.25 Å and then quantify their kinetics of incorporation. Kinetic measurements demonstrated that the high-fidelity bacteriophage T7 DNA polymerase displays high selectivity for the overall size of a base pair during the polymerization cycle (Kim et al., 2006). In particular, incorporation efficiency decreased ∼300-fold with nucleobase-modified base pairs that are 0.4 Å larger than the optimum size of a natural pair (10.6 Å). Furthermore, nucleobase-modified base pairs that are 0.3 Å smaller were formed with reduced catalytic efficiencies compared to normal base pairs (Kim et al., 2006). Collectively, these data suggested that active site “tightness” of a DNA polymerase is an important determinant for controlling replicative fidelity.

To further interrogate this model, the Kool laboratory also designed and tested 4-methylbenzimidazole as a complementary partner for 2,4-difluorotoluene (Figure 2C). This base pair is proposed to be geometrically identical to an A:T base pair as the methyl group of 4-methylbenzimidazole is similar in size to the amino group of adenine while the fluoro groups of 2,4-difluorotoluene are comparable in size to the keto oxygens of dTTP. Consistent with this model, kinetic studies showed that 2,4-difluorotoluene is incorporated opposite 4-methylbenzimidazole with only a ∼200-fold lower efficiency compared to the insertion of dTTP opposite A (Guckian et al., 1998).

To further optimize polymerization efficiency, 4-methylbenzimidazole was modified slightly to generate 9-methylimidazo [(Kool, 2002; Kool and Sintim, 2006)-b]pyridine (denoted as dQ) (Morales and Kool, 1998). dQTP is inserted opposite a templating 2,4-difluorotoluene with an efficiency equal to that for inserting dATP opposite 2,4-difluorotoluene (Morales and Kool, 1998). In addition, the E. coli Klenow fragment can extend beyond dQ when it is paired opposite 2,4-difluorotoluene with a∼300-fold greater efficiency compared to extending beyond 4-methylbenzimidazole paired opposite 2,4-difluorotoluene (Morales and Kool, 1998). As discussed later, the ability of a DNA polymerase to extend beyond nucleobase-modified base pairs lacking conventional hydrogen bonding groups is an important step in efforts to reengineer DNA.

**FIGURE 3:**
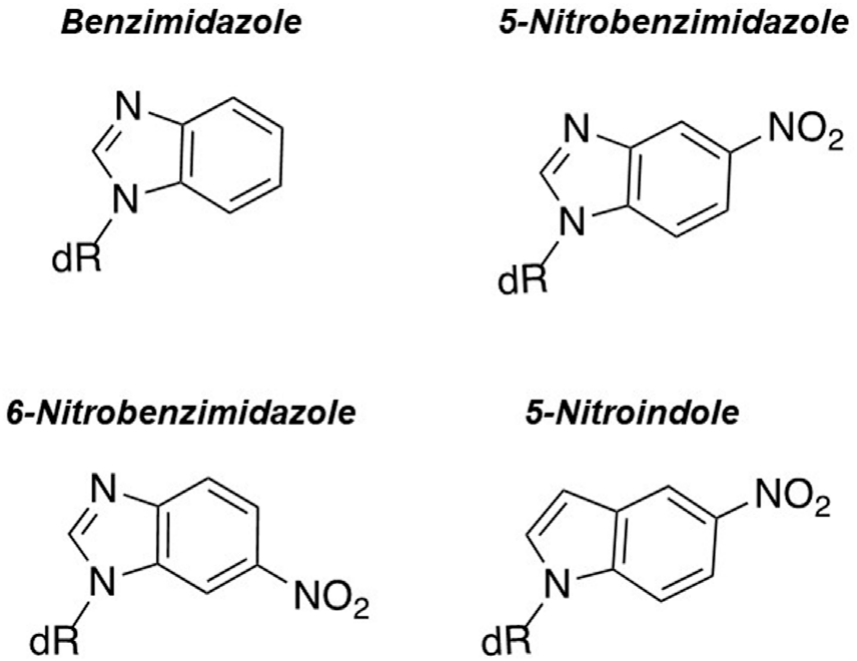
Structures of benzimidazole, 6-nitrobenzimidazole, 5-nitrobenzimidazole, and 5-nitroindole.

Other research groups have used different nucleobase-modified nucleotides to further explore the underlying mechanism of nucleotide selection and polymerization fidelity in the absence of hydrogen bonds. For example, the Kuchta laboratory quantified the incorporation of purine analogs such as benzimidazole, 5-nitrobenzimidazole, 6-nitrobenzimidazole, and 5-nitroindole (Figure 3) (Chiaramonte et al., 2003). Their studies showed that eukaryotic pol α and E. coli Klenow fragment utilize these nucleotide analogs with efficiencies that are only 10-fold lower than that for forming a natural base pair (Moore et al., 2004; Kincaid et al., 2005). With pol α, the presence of a nitro group appears to increase binding affinity for this class of analog as the K_m_ values for 5-nitrobenzimidazole, 6-nitrobenzimidazole, and 5-nitroindole are lower compared to benzimidazole. In addition, the K_m_ values for these particular nitro containing analogs are only 10-fold higher than K_m_ values for natural dNTPs.

Surprisingly, an opposite trend is observed with the Klenow fragment as the measured K_m_ values for nitro-containing analogs are ∼100-fold higher compared to natural dNTPs. However, the most striking result is that the Klenow fragment preferentially inserts these analogs opposite purines rather than pyrimidines (Moore et al., 2004; Kincaid et al., 2005) which is noteworthy since it is counter to predictions set by the “shape complementarity” model. In general, while these data again recapitulate that hydrogen bonding interactions are not absolutely required for DNA polymerization, they suggest that the “steric fit” model cannot be universally applied to all DNA polymerases with all nucleobase-modified nucleotide substrates.

Based on these and other experimental data, the Kuchta laboratory proposed a model invoking “negative selection” as the predominant force used to maintain replication fidelity (Kincaid et al., 2005). Implicit in this model is the presence of a general binding step that allows a DNA polymerase to initially sample all dNTPs with equal affinity. During this sampling step, the nucleobase of the dNTP interacts with the templating base, and if the molecular interactions are favorable, the base pair adopts the lowest free energy conformation. When this criterion is met, the polymerase proceeds with a conformational change step that occurs prior to chemistry. This conformational change step is crucial as it locks the incoming dNTP within the active site of the DNA polymerase. In contrast, attempts to form an energetically unfavorable basepair are hindered during this conformational change step. This prevents nucleotide incorporation which then allows the incorrect nucleotide to dissociate from its active site rather than be misincorporated. The inferred biological advantage of this mechanism is that a DNA polymerase can rapidly sample various nucleotides while only accepting the dNTP that adopts the lowest free energy conformation.

**FIGURE 4:**
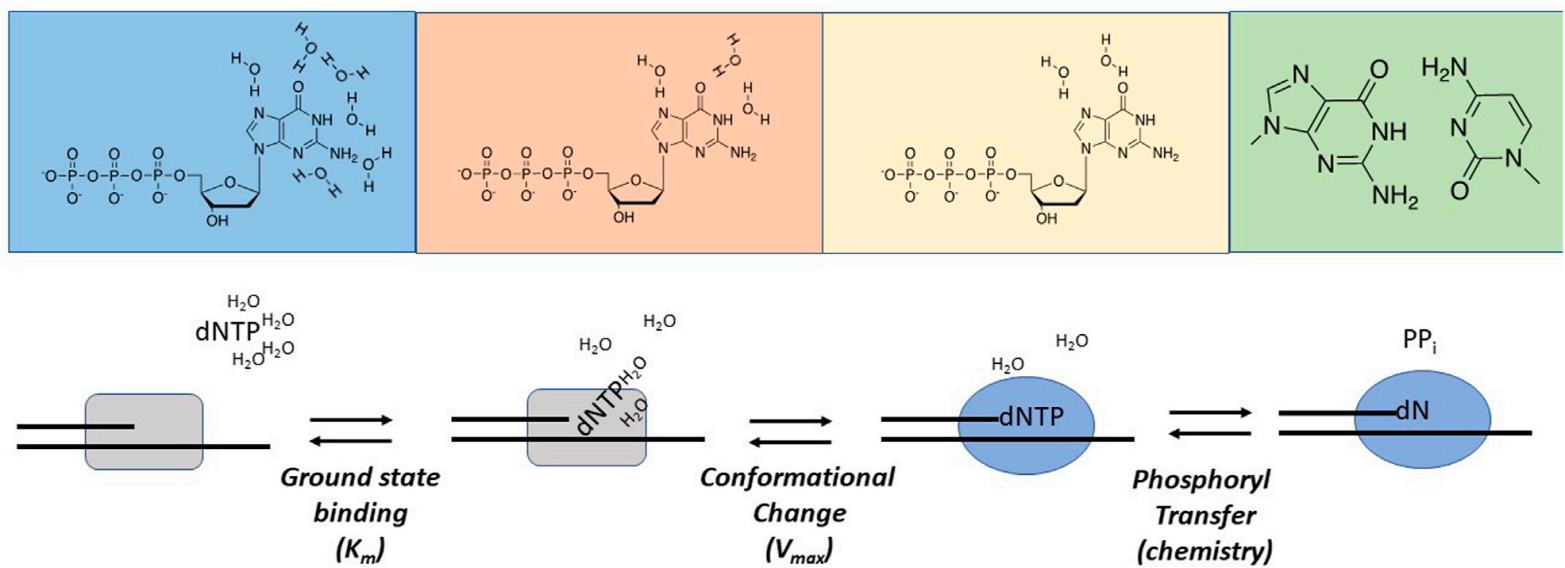
Hypothetical nucleobase desolvation mechanism catalyzed by DNA polymerases. See text for complete details.

Examining Nucleobase Desolvation During Replication. Nucleobase desolvation plays an essential role during replication as water molecules surrounding the functional groups of the incoming dNTP must be removed prior to forming hydrogen bonds within the interior of the DNA helix. Goodman and colleagues were among the first to evaluate the role of nucleobase desolvation as a determinant for maintaining polymerization fidelity (Petruska et al., 1986). Thermodynamic studies of duplex DNA in aqueous solution demonstrated that free energy differences between correct and incorrect base pairs range between 1 and 3 kcal/mol (Petruska et al., 1988). Based on these values, it was predicted that DNA polymerases would display only a 5- to 150-fold discrimination against incorporating an incorrect nucleotide. However, these predicted discrimination factors are substantially lower than the observed frequencies of nucleotide misincorporation events measured with most DNA polymerases (Fersht et al., 1982; Goodman and Fygenson, 1988; Kunkel and Bebenek, 2000). To reconcile this dichotomy, Goodman and colleagues performed a comprehensive thermodynamic analysis of melting temperatures for duplex DNA containing matched and mismatched template-primer termini. These values were then compared with kinetic data measuring the incorporation of correct and incorrect nucleotides using identical DNA sequences. Differences between data sets were interpreted with respect to a model integrating ucleobase desolvation as a key determinant in polymerization fidelity. In this model, water surrounding the functional groups of the incoming dNTP must be displaced as it enters the active site of DNA polymerase in order for hydrogen bonding and base-stacking interactions to occur within the interior of the double helix (Figure 4). The consequence of water expulsion is that base stacking and hydrogen-bonding interactions become amplified within the active site of the DNA polymerase, and the associated differences in free-energy are sufficient to account for the high degree of replicative fidelity.

**FIGURE 5:**
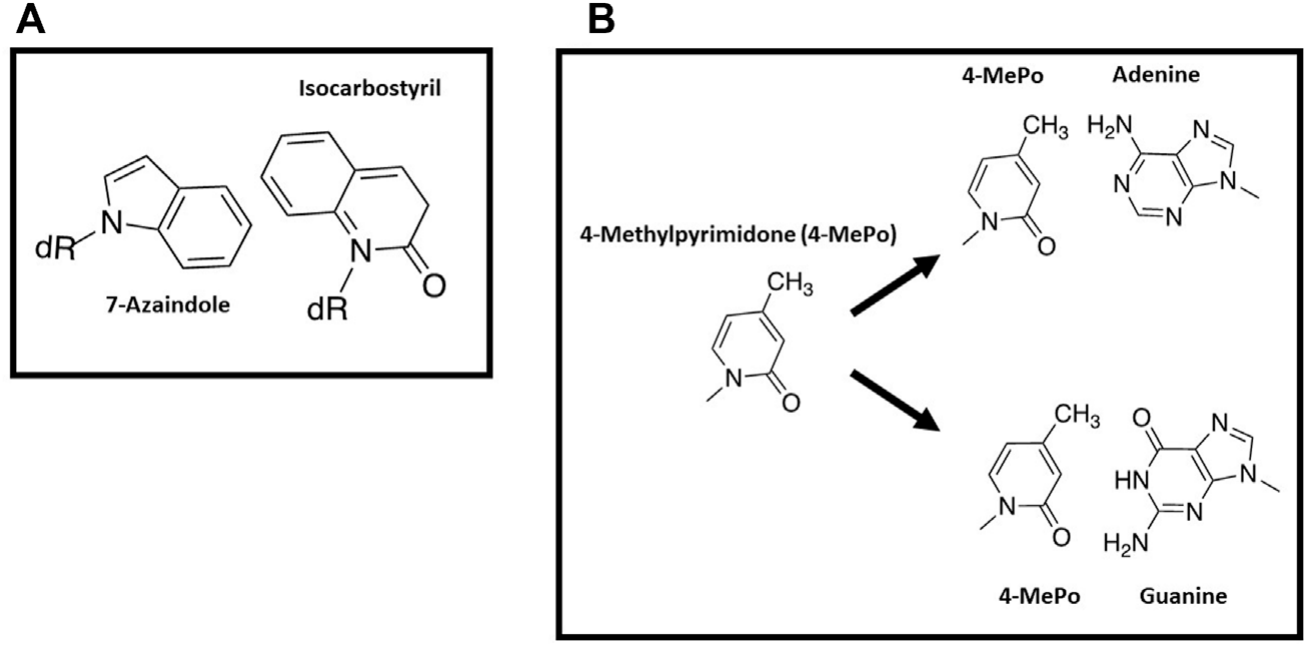
**(A)** Structures of 7-azaindole and isocarbostyril nucleosides **(B)** Structure of 4-methyl-pyridone and pairing interactions opposite adenine or guanine.

With this background, several groups have used nucleobase-modified nucleotides as innovative probes to further interrogate the influence of nucleobase desolvation during DNA polymerization. For example, studies by Romesberg and Schultz (Wu et al., 2000) examined basepairs formed between 7-azaindole and isocarbostyril nucleosides (Figure 5A). Despite a lack of hydrogen bonding potential on either nucleobases, isocarbostyril triphosphate is incorporated opposite 7-azaindole rather efficiently as the k_cat_/K_m_ is only 100-fold lower compared to the incorporation of dTTP opposite A. In addition, replication of the nucleobase-modified base pair is kinetically symmetrical as the efficiency for incorporating 7-azaindole triphosphate opposite isocarbostyril is nearly identical to the incorporation of isocarbostyril opposite 7-azaindole (Wu et al., 2000). In general, the facile incorporation of a hydrophobic analog opposite a non-complimentary partner suggests that hydrophobicity and pi-electron stacking interactions can compensate for a lack of hydrogen bonding interactions.

Another example highlighting this phenomenon comes from the Hirao lab examining the incorporation of 4-methylpyrimid-2-one triphosphate (4-MePoTP) opposite natural nucleobases (Figure 5B) (Hirao et al., 2004). This pyrimidine analog was designed to be a selective pairing partner for G rather than A since the 4-methyl group should sterically clash with the 6-amino group of adenine. While the 3-hydrogen of 4-methylpyrimid-2-one is predicted to collide with the 1-imino proton of guanine, the interactions between the two bases were proposed to be favorable due to hydrogen-bonding interactions of the 2-keto group of 4-methylpyrimid-2-one with the 2-amino group of G. Kinetic data obtained using the E. coli Klenow fragment is consistent with this design since only dGTP is incorporated opposite 4-methylpyrimid-2-one as the templating base (Hirao et al., 2004). However, 4-methylpyrimid-2-one triphosphate is unusually promiscuous as it is incorporated opposite all four natural templating nucleobases (Hirao et al., 2004). As discussed below, it is possible that the hydrophobic nature of 4-methylpyrimid-2-one makes it easier to strip water molecules away from modified nucleobase, thus increasing its promiscuous utilization.

Nucleobase Desolvation and pi-Stacking Interactions During the Replication of Damaged DNA. DNA is highly susceptible to a large number of modifications that can directly influence hydrogen bonding interactions. To date, hundreds of distinct DNA lesions have been identified in both prokaryotic and eukaryotic cells (reviewed in (Bauer et al., 2015)). While both species possess several pathways to correct damaged DNA, there are instances in which DNA lesions escape repair and are subsequently misreplicated (Goodman and Woodgate, 2013; Vaisman and Woodgate, 2017; Yang and Gao, 2018). The ability of a DNA polymerase to incorporate nucleotides opposite and beyond damaged DNA is a process called translesion DNA synthesis (TLS).

**FIGURE 6:**
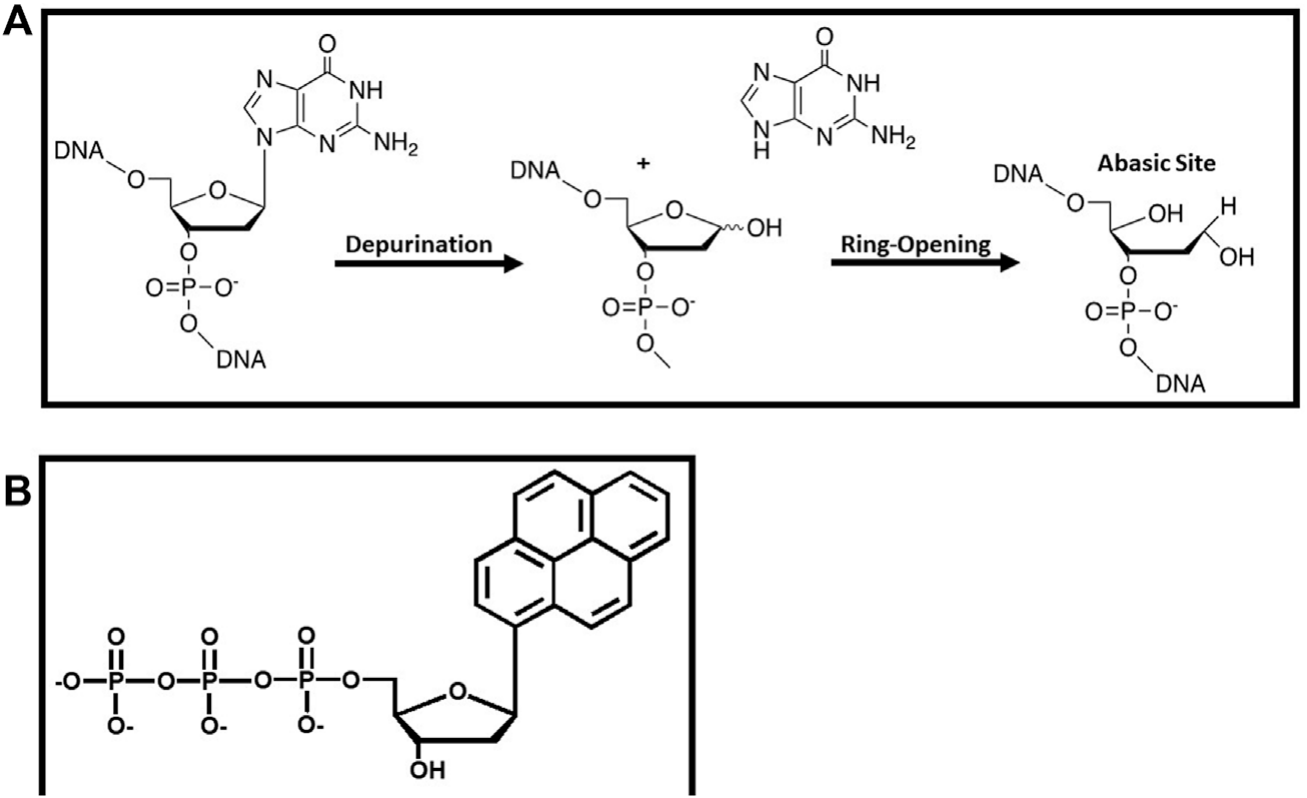
**(A)** Formation and structure of an abasic site that functions as a non-instructional DNA lesion **(B)** Structure of pyrene triphosphate used to probe the replication of an abasic site.

Perhaps the most commonly formed DNA lesion is an abasic site which is generated by the hydrolysis of the glycosidic bond between the C1′ of ribose and the N9 of a purine or the N1 of a pyrimidine (Figure 6A). Abasic sites are classified as “non-instructional” DNA lesions since coding information is lost during this depurination event. Despite the lack of coding information, a large number of DNA polymerases including eukaryotic pol α (Shibutani et al., 1997) and pol δ (Mozzherin et al., 1997), the E. coli DNA polymerase I (Paz-Elizur et al., 1997), the bacteriophage T4 DNA polymerase (Berdis, 2001), and HIV reverse transcriptase (Cai et al., 1993) preferentially insert dAMP opposite this lesion. This kinetic phenomenon is enigmatic since the ability of a DNA polymerase to preferentially incorporate a specific nucleotide opposite a non-instructional lesion cannot be reconciled by models invoking hydrogen-bonding interactions.

Several research groups have used nucleobase-modified nucleotides to probe the mechanistic basis for the preferential incorporation of dAMP opposite an abasic site. One of the earliest endeavors was reported by the Kool laboratory investigating the role of shape complementarity (Matray and Kool, 1999). Their work demonstrated that the E. coli Klenow fragment incorporated pyrene triphosphate (Figure 6B) opposite an abasic site ∼100-fold more efficiently than any of the four natural dNTPs (Matray and Kool, 1999). The increased efficiency reflects a higher binding affinity for the analog coupled with a faster rate constant for its incorporation. The facile insertion of pyrene triphosphate opposite this lesion was interpreted within the context of the shape complementarity model as the “void” present at an abasic site could be easily filled by the bulky nucleobase. Indeed, modeling studies showed that the shape and size of a pyrene:abasic site mispair is nearly identical to that of a natural A:T basepair (Matray and Kool, 1999). However, it is clear from the structure of pyrene that other biophysical properties such as pi-stacking and increased nucleobase hydrophobicity may also play important roles.

**FIGURE 7:**
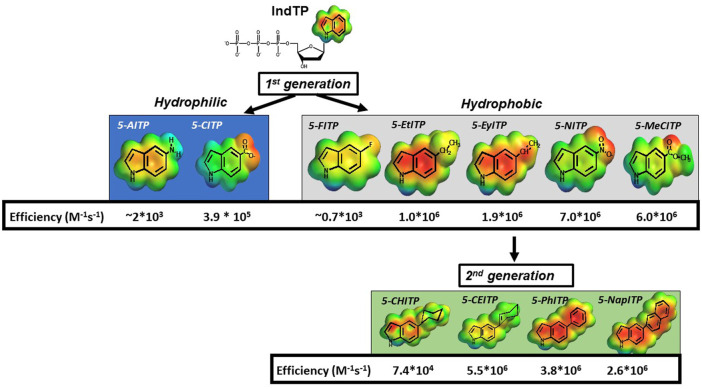
Structures of various 5-substituted indolyl deoxynucleotides tested as nucleotide substrates for DNA polymerase during the replication of an abasic site.

Our contributions in this area have examined the roles of pi-electron density and nucleobase desolvation during the replication of structural diverse DNA lesions. The approach was to quantify the enzymatic insertion of modified indolyl 2′-deoxyribose nucleotides opposite an abasic site to develop a structure-activity relationship for these features (Figure 7). Indole was initially used as a molecular scaffold as this represents the core structure of dATP, the preferred natural nucleotide substrate. First generation analogs were produced by introducing small functional groups at the 5-position of indole and are classified as hydrophilic (-NH_2_ and -COO^-^) or hydrophobic (-F, -CH_2_CH_3_, -CHCH_2_, -COOCH_3_, and -NO_2_). Kinetic studies showed that hydrophilic analogs were incoprorated opposite an abasic site with lower overall efficiencies compared to most hydrophobic analogs (5-Et-ITP, 5-EyITP, 5-MeCITP, and 5-NITP) (Reineks and Berdis, 2004; Zhang et al., 2004; Zhang et al., 2005a; Zhang et al., 2006a; Motea et al., 2013). Furthermore, hydrophobic analogs that possess pi-electron density display remarkably high efficiencies for insertion opposite this lesion. In particular, 5-NITP is inserted opposite an abasic site with approximately 1,000-fold greater efficiency compared to dATP (Reineks and Berdis, 2004). Remarkably, the fast k_pol_ value of 126 s^−1^ and low K_d_ value of 18 μM are nearly identical to those measured for the incorporation of dATP opposite T (Capson et al., 1992). Collectively, these data reiterate that hydrogen bonding interactions are not essential for nucleotide insertion. However, the facile incorporation of these small analogs is not consistent with models invoking steric fit or shape complementarity but rather highlight the importance of desolvation and pi-electron stacking.

To further interrogate this hypothesis, second generation analogs were synthesized and tested for insertion opposite this lesion (Zhang et al., 2006b; Motea et al., 2011). These analogs containing cyclohexyl, cyclohexene, and phenyl moieties are similar in shape, size, and hydrophobicity (Figure 7). However, the primary difference is the amount of pi-electron density dictated by the absence and presence of double bonds. As indicated, the overall catalytic efficiency for analogs possessing pi-electron density (5-CEITP and 5-PhITP) are greater than that measured for 5-CHITP which lacks significant pi-electron density at the 5-position of the indole. These data are important as they again reinforce the concept that nucleobase desolvation and pi-electron density are important factors for efficient insertion opposite non-instructional DNA lesions. However, the efficiency for inserting 5-CEITP and 5-PhITP are still lower than that measured for 5-NITP which is significantly smaller than these two analogs. This result infers that steric fit and shape complementarity play minimal roles during TLS.

**FIGURE 8:**
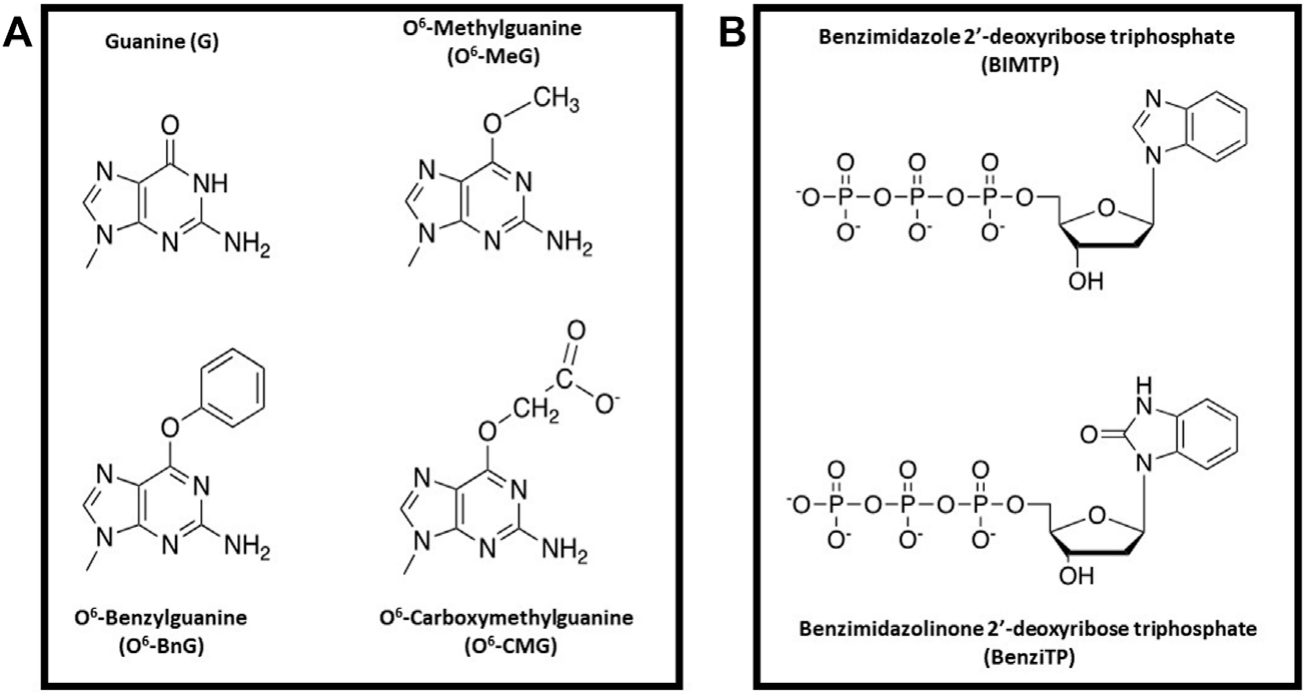
**(A)** Structural comparison of alkylated-guanine DNA lesions (O^6^MeG, O^6^BnG, and O^6^CMG) with G **(B)** Structures of BIMTP and BenziTP used to probe the replication of O^6^BnG.

Nucleobase-modified nucleotides selective for different DNA lesions have also been developed by other research groups (Livingston et al., 2008; Gahlon et al., 2013; Stornetta et al., 2013; Wyss et al., 2016; Fleming et al., 2017). One interesting example is work from the Sturla group examining the replication of O^6^-alkylG DNA adducts generated by alkylation of the exocyclic oxygen at the 6-position of deoxyguanosine. These include lesions such as O^6^-methylguanine (O^6^MeG), O^6^-benzylguanine (O^6^BnG), and O^6^-carboxymethyl guanine (O^6^CMG) (Figure 8A). In many cases, the cellular consequences caused by these lesions can best be described as a “double-edged sword”. For example, methyl nitrosamines which is present in tobacco products can generate a number of O^6^-alkylG DNA adducts, and their misreplication can induce mutagenesis to initiate carcinogenesis (Eadie et al., 1084). On the other hand, treatment with the chemotherapeutic drug, temozolomide, can form similar lesions that produce beneficial cell-killing effects against several types of cancer (Weller et al., 2005). Finally, O^6^-CMG is a unique lesion arising from endogenous nitrosylation of glycine and subsequent reaction with DNA (Kino et al., 2020).

A goal of the Sturla group is to develop nucleobase-modified nucleotides that can selectively replicate and amplify genomic DNA containing these lesions. This is an important endeavor since O^6^MeG is a miscoding DNA lesion while both O^6^BnG and O^6^CMG tend to block DNA polymerases used in PCR. To address this problem, the Sturla group developed a perimidinone analog that, when incorporated into DNA, increases the thermostability on nucleic acids containing O^6^BnG lesion better than those containing G (Stornetta et al., 2013). Unfortunately, this stabilization is not caused by forming a “conventional” base pair but rather through intercalation of the analog into duplex DNA caused by increased base stacking interactions (Kowal et al., 2013).

To combat this problem, nucleobase-modified nucleotides designated as BIMTP and BenziTP were synthesized and tested as substrates for DNA polymerases when replicating DNA containing either O^6^BnG or G (Figure 8B) (Trantakis and Sturla, 2014). Kinetic studies showed that the specialized DNA polymerase, Dpo4, incorporated BenziMP opposite O^6^BnG at higher levels compared to G. These results were interpreted to reflect positive interactions of the H-bond acceptor present on O^6^BnG with the N–H donor on Benzi. In contrast, the G:Benzi base pair has unfavorable steric interactions between the hydrogen bond donor N–H on G with the N–H donor on Benzi. Additional studies were performed using a mutant form of KlenTaq designated KTqM747K. This DNA polymerase is roughly 2-fold more proficient at incorporating BenziMP opposite O^6^-BnG compared to wild-type KlenTaq. In addition, the KTqM747K polymerase incorporated BenziMP approximately 25-fold more efficiently opposite O^6^BnG compared to G. This selectivity is not due to a global reduction in replicative fidelity as this mutant polymerase incorporates dCMP opposite O^6^BnG ∼30,000-fold less efficiently compared to G. Finally, kinetic studies demonstrated that BenziMP paired opposite O^6^BnG can be elongated when supplied with natural nucleotides. This is an important achievement since replication beyond O^6^-BnG is typically stalled when BenziTP is omitted from the reaction.

## Using nucleobase-modified nucleosides in synthetic biology

### Attempts to expand the genetic code

This next section describes how mechanistic information obtained using nucleobase-modified nucleotides has been applied in efforts to re-design nucleic acid. For example, rationally designing new base-pairing partners could produce unique biological polymers that function as universal primers for PCR amplification (Johnson et al., 2004), biosensors (Ting et al., 2004), and nanowires (Bang et al., 2005). Perhaps the most challenging effort, however, involves creating a “third” base pair to expand the existing genetic code so that additional coding information could be used for the biosynthesis of unique proteins containing novel amino acids (Singh et al., 2021a). This capability has incredible potential for new biotechnological applications ranging from increasing the thermostability of proteins to expanding the chemical capabilities of existing enzymes.

While it is possible to transiently produce proteins bearing non-native amino acids, creating an expanded and stable genetic code for sustained biological function is a daunting task since it requires an integrated approach to first generate a re-coded genome and then introduce new biomolecules that are needed for efficient transcription and translation. In addition, it is essential that these components work seamlessly so that the efficiency of other biological pathways such as protein folding, post-translational modifications, and proteolytic degradation are not adversely affected. While these are important issues, the sections below focus on certain efforts that highlight how nucleobase-modified nucleotides are used in synthetic biology.

### Altering hydrogen-bonding interactions

**FIGURE 9:**
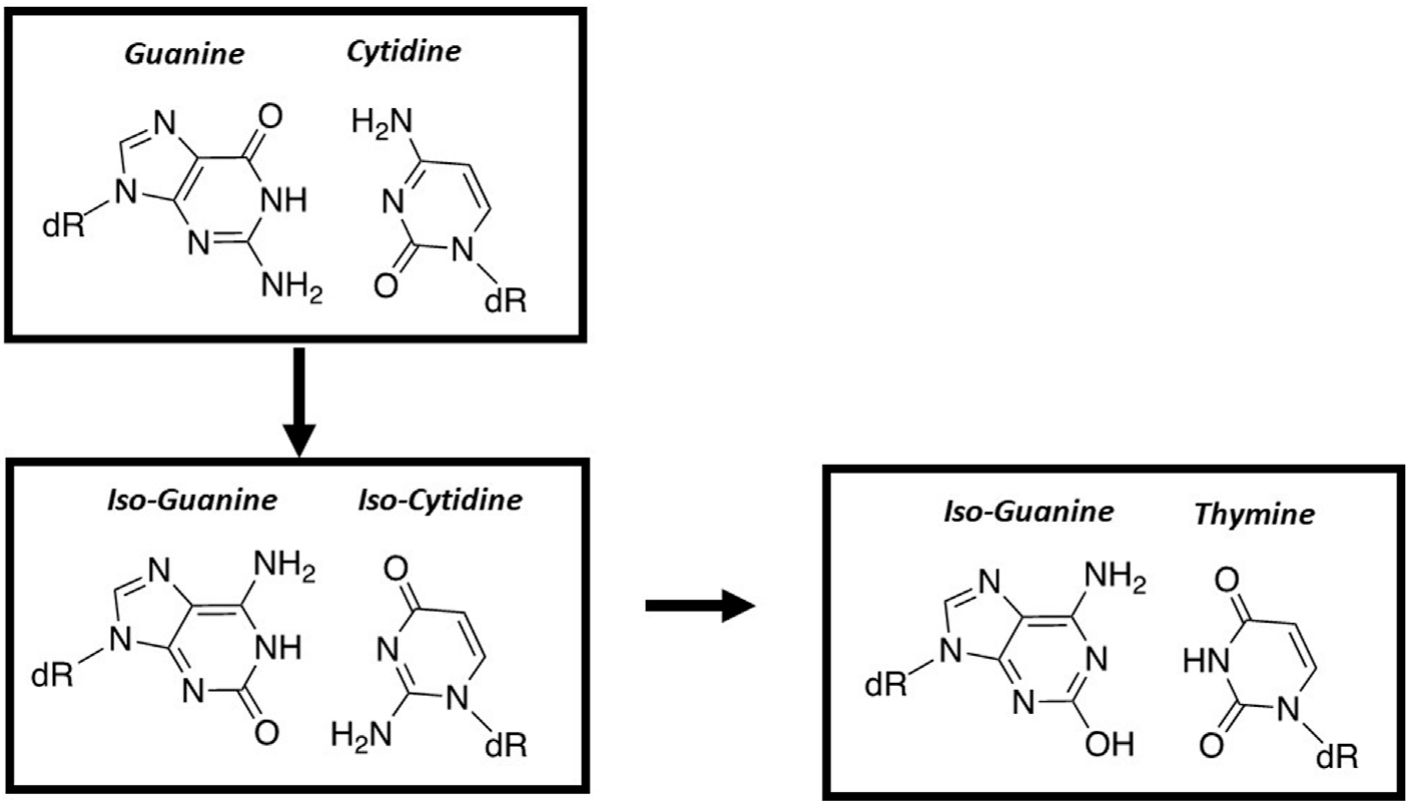
Structural comparison of nucleobase-modified base pairs composed of iso-guanine and iso-cytosine with the natural G:C base pair.

The first published work to rationally design a “third base-pair” was reported by Steve Benner’s group (Piccirilli et al., 1990). These base pairs include 5-(2,4-diaminopyrimidine:xanthosine base and iso-cytosine:iso-guanine (Figure 9). Benner’s group demonstrated that the E. coli Klenow fragment can efficiently form an iso-cytosine:iso-guanine base pair (Horlacher et al., 1995). Unfortunately, the overall fidelity of this new base pair is low since iso-guanine can be easily incorporated opposite a templating T and *vice versa* (Horlacher et al., 1995). This capability reflects facile tautomerization of iso-guanine from the expected keto to the enol form.

### Developing hydrophobic nucleosides as novel pairing partners

**FIGURE 10:**
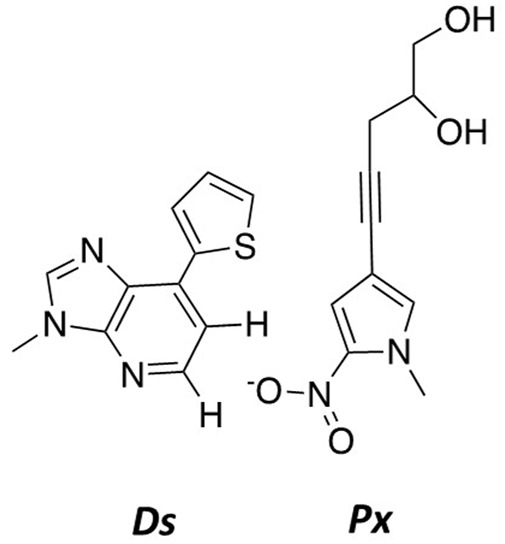
Structures of Ds and Px.

Another important example is work by the Hirao group that pioneered the development of several nucleobase-modified nucleotides by merging strategies of altered hydrogen-bonding patterns reinforced by exclusion using steric-hindrance (Ohtsuki et al., 2001; Hirao et al., 2002). By combining shape complementarity (positive selection) with steric and electrostatic exclusion (negative selection), they were able to generate a novel hydrophobic base pair designated as Ds:Px (Figure 10) (Kimoto et al., 2009; Yamashige et al., 2012; Okamoto et al., 2016). A key feature of this strategy is the enhancement in fidelity by minimizing pairing interactions of the nucleobase-modified nucleotide with natural nucleobases. Based on this success, the Hirao group generated a new hydrophobic base analog by removing the 2-amino group and replacing the 1-nitrogen with carbon. Despite lacking hydrogen bonding interactions, the Ds–Pa pair was the first nucleobase-modified base pair reported to function in PCR and transcription as a third base pair (Kimoto et al., 2016).

To further improve this base pair, the Hirao group modified Pa to increase its pairing efficiency while decreasing the possibility for forming mixed mispairs. This was achieved by generating the analog designated Px by adding a propynyl group to position 4 of the pyrrole group of Pa as well as replacing the aldehyde group with a nitro group (Sefah et al., 2014). The propynyl group increases the affinity to DNA polymerases through stacking interactions within its active site, and this reinforces pairing between Ds–Px. In contrast, the nitro group is proposed to electrostatically repel the 1-nitrogen of A to prevent forming mispairs such as A opposite Px.

The Hirao group subsequently used these nucleobase-modified nucleotides to produce novel aptamers that bind to a specific target molecule with high affinity. Aptamers are often generated using a technique called systematic evolution of ligands by exponential enrichment (SELEX) that combines combinatorial approaches with molecular biology methods to produce an oligonucleotide library for screening. The Hirao group expanded this technique to develop a new SELEX method coined Expansion for SELEX (ExSELEX) using a DNA library containing Ds. The purpose of including Ds was to expand and binding interactions and increase binding affinities by introducing a hydrophobic nucleobase with the four natural hydrophilic nucleobases. Using the ExSELEX procedure, they generated three Ds-DNA aptamers that bound several therapeutically important proteins including vascular endothelial growth factor 165 (VEGF_165_), interferon-γ (IFNγ), and von Willebrand factor A1-domain with high affinities ranging from K_d_ values of 1–75 p.m. (Knudsen et al., 2002; Matsunaga et al., 2015; Matsunaga et al., 2017). Binding affinities for these Ds-containing aptamers are significantly higher compared to conventional DNA aptamers containing only natural nucleobases. It is important to note that these particular aptamers contain at least two Ds bases, and their presence is critical for their higher binding affinity (Matsunaga et al., 2015; Matsunaga et al., 2017).

### Creating novel DNA polymerases

In general, there are three major pitfalls associated with developing an expanded genetic code. As mentioned previously, one complication is the tendency for nucleobase-modified nucleotides to readily form self-pairs as opposed to forming unique complementary base-pairing combinations. The second challenge is to prevent excision of the formed base pair by exonucleolytic proofreading activity possessed by most high-fidelity DNA polymerases. This complication is discussed more with respect to the potential therapeutic activity of nucleobase-modified nucleosides. In addition, there are several outstanding review articles that describe chemical approaches to increase the stability of oligonucleotides that contain modified nucleotides (Knudsen et al., 2002; Stovall et al., 2014; Du et al., 2016). The third and final pitfall has been to develop a novel base pair that can be easily extended by a single DNA polymerase. Work published by Romesberg and colleagues highlights these challenges but also provide innovative approaches to combat these problems. As previously described, the Romesberg group demonstrated that the E. coli Klenow fragment can form a 7-azaindole:7-azaindole self-pair but is unable to extend it efficiently (Wu et al., 2000). In contrast, the eukaryotic DNA polymerase β is unable to synthesize the self-pair but is able to extend the 7-azaindole:7-azaindole self-pair as efficiently as a natural base pair (Wu et al., 2000). Taking advantage of these opposing activities, they developed a binary DNA polymerase system using the E. coli Klenow fragment to first enzymatically form the novel base pair and then used the eukaryotic DNA polymerase β to extend the self-pair to synthesize full length DNA containing 7-azaindole in the template strand. While cumbersome, this represents an important milestone toward expanding the genetic code as it demonstrates the ability to form and extend an nucleobase-modified-natural base pair efficiently and with high fidelity.

Based on this success, other groups have used molecular cloning techniques to re-engineer into a single DNA polymerase to possess both enzymatic activities. Perhaps the strongest evidence for this approach is work published by the groups of Loakes and Holinger who developed a unique strategy to rapidly evolve DNA polymerases capable of incorporating and extending beyond nucleobase-modified nucleotides. The technique, called “compartmentalized self-replication” (CSR), is based on a feedback loop in which a mutant DNA polymerase can only replicate its own gene (Tawfik and Griffiths, 1998; Ghadessy et al., 2001). This is possible since the polymerization reaction is compartmentalized into the aqueous phase of a water-in-oil emulsion (Stovall et al., 2014). This compartmentalization provides a single, self-replicating system from potentially thousands of other mutant DNA polymerases contained within their own individual water-in-oil emulsions. This closed system also allows for adaptive gains which are used to genetically amplify the gene encoding for the DNA polymerase containing the desired function.

Using this CSR system, d'Abbadie et al. (2007) used directed evolution to first generate a library of mutant DNA polymerases which was then screened to identify a unique mutant DNA polymerase capable of replicating hydrophobic base analogs with higher efficiency compared to wild type DNA polymerases. Their approach used molecular breeding of the polA genes from three members of the genus Thermus (Taq (T. aquaticus), Tth (T. thermophilus), and Tfl (T. flavus)) (Du et al., 2016). In addition, they used flanking primers containing two nucleobase-modified nucleobases, 5-nitroindole (5-NI) and 3-carboxamide-5-nitroindole (5NIC). Using CSR selection reactions, they isolated a unique DNA polymerase, designated 5D4, that displayed improved abilities to incorporate and extend beyond various hydrophobic base analogs such as 7-azaindole that form self-pairs as well as several nucleobase-modified nucleotides. In particular, the 5D4 polymerase can efficiently by-pass 5NI and 5NIC which allows for PCR of amplification of DNA using modified primers with 5NIC paired opposite abasic sites. This is a noteworthy accomplishment since both 5-NI and 5NIC significantly stall PCR reactions since they are poorly bypassed by most DNA polymerases. In addition, sequencing of the generated PCR products was used to interrogate the coding potential of 5-NI and 5NIC when replicated by the 5D4 DNA polymerase. Surprisingly, 5-NI as the templating base directed the incorporation of dAMP approximately ∼90% of the time. In contrast, 5NIC as the templating base showed preferentially incorporation of dTMP ∼75% of the time whereas dAMP, dGMP and dCMP were incorporated less frequently.

The 5D4 DNA polymerase also displayed superior activity compared to other DNA polymerases using nucleobase-modified analogs such as pyrene and 5-NI paired opposite an abasic site. Typically, these nucleobase-modified nucleotides are refractory to elongation by natural DNA polymerases (Matray and Kool, 1999; Reineks and Berdis, 2004). However, the 5D4 DNA polymerase efficiently extends beyond both 5-NI and pyrene when paired opposite this non-instructional DNA lesion. In fact, it is remarkable that the 5D4 DNA polymerase extends beyond a pyrene:abasic site pair more efficiently that a dAMP:abasic site base pair. Collectively, the ability to form and effectively extend various base pairs containing nucleobase-modified nucleotides such as pyrene and 5-NI raises the potential to synthesize long polymers containing these and other nucleobase-modified nucleobases to re-engineer the genetic code.

## Nucleobase-modified nucleosides as potential therapeutic agents

### Cancer and chemotherapy

**FIGURE 11:**
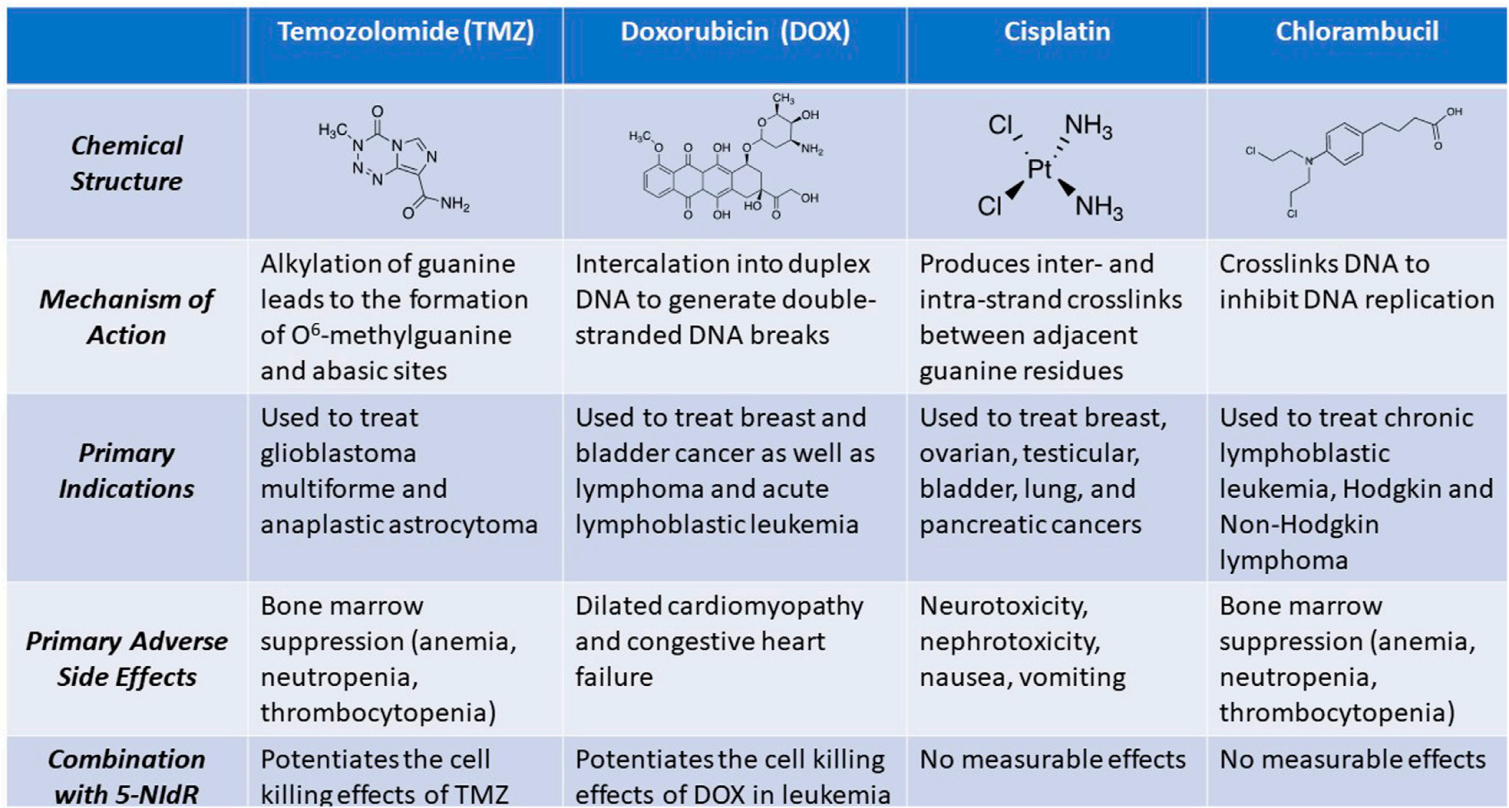
Structures and associated activities of representative anti-cancer agents that primarily function as DNA damaging agents.

Cancer currently ranks as the second leading cause of death in the United States and other industrialized nations (Siegel et al., 2022). For most solid cancers, standard treatments begin by reducing tumor burden *via* surgery and/or ionizing radiation therapy. This is then followed by several rounds of chemotherapy designed to eliminate cancer cells that remain after these procedures. Many chemotherapeutic agents target nucleic acid metabolism by modifying the functional groups present on the four natural nucleobases that compose DNA (Figure 11). As mentioned earlier, the drug temozolomide produces several distinct DNA lesions including N^3^-methyladenine, O^6^-methylguanine, and N^7^-methylguanine that all cause cytostatic and cytotoxic effects (Roos and Kaina, 2013). Methylation at the N7 position of guanine is particularly important as this modification enhances spontaneous depurination to produce an abasic site which due to its non-instructional nature is a highly toxic DNA lesion (Glaab et al., 1999). Unfortunately, many cancers have increased TLS activity caused by overexpression of distinct DNA polymerases that efficiently replicate these DNA lesions. Unregulated TLS activity can cause mutagenesis and generate drug resistance to anti-cancer agents like temozolomide that damage DNA (Zhou et al., 2013; Tomicic et al., 2014; Choi et al., 2018a; Singh et al., 2021b; Stanzione et al., 2022). An approach that we’ve developed to combat these problems is to use nucleobase-modified nucleotides to selectively inhibit the DNA polymerases involved in replicating DNA lesions generated by drugs that damage DNA (Choi et al., 2017).

### Cell-based studies testing the efficacy of nucleobase-modified nucleosides

As a first step toward this goal, we tested the ability of the nucleobase-modified nucleotide analogs displayed in Figure 11 to serve as substrates for human DNA polymerases capable of replicating abasic sites. Our *in vitro* studies identified 5-NITP as a unique analog that is efficiently and selectively inserted opposite this DNA lesion by two high-fidelity DNA polymerases (pol δ and pol ε) and two specialized DNA polymerases (pol η and pol ι (Choi et al., 2018b). In addition, 5-NITP is poorly inserted opposite unmodified DNA (A, C, G, or T). Furthermore, we found that the nucleobase-modified analog is refractory to elongation and thus functions as an effective chain terminator that blocks TLS activity. The chain termination activity is unique since the analog does not possess modifications to the deoxyribose moiety similar to other chain terminating nucleoside analogs such as AZT and fludarabine. In addition to possessing unique chain termination capabilities, 5-NI is removed from opposite the DNA lesion much more slowly compared to natural dNTPs paired opposite the abasic site (Zhang et al., 2005b). The rate of enzymatic excision becomes significantly faster when 5-NI is paired opposite natural templating nucleobases such as T and C (Zhang et al., 2005b). Furthermore, idle turnover measurements, i.e., the repetitive process of inserting 5-NITP and removing it from DNA, demonstrate that the nucleobase-modified nucleotide is more stable opposite an abasic site compared to natural dNTPs (Zhang et al., 2005b). Reduced idle turnover of 5-NITP reflects favorable insertion kinetics combined with reduced exonuclease-proofreading capacity. Collectively, these results indicate that the nucleobase-modified nucleotide selectively and efficiently blocks the replication of an abasic site.

Cell-based studies next evaluated if the corresponding nucleoside, 5-NIdR, increases the cytotoxicity of temozolomide by inhibiting TLS activity. Promising results were obtained using the human cell line, U87, as a model for glioblastoma multiforme (GBM), a deadly form of brain cancer. We first confirmed that U87 cells are resistant to temozolomide as the measured LD_50_ value for the DNA damaging agent is greater than 100 μM (Choi et al., 2018b). In addition, 5-NIdR displays low potency as the LD_50_ value is greater than 100 μg/ml. In this case, the low potency of 5-NIdR is expected since our mechanistic studies showed that the corresponding nucleoside triphosphate, 5-NITP, is poorly incorporated opposite undamaged DNA. However, combining 5-NIdR with temozolomide produces a robust cell killing effect. In particular, this combination generates significantly higher levels of early and late stage apoptosis compared to U87 cells treated with identical concentrations of temozolomide or 5-NIdR used individually.

The ability of 5-NIdR to increase the cell killing effects of temozolomide was further confirmed using flow cytometry to monitor cell cycle progression as a function of drug treatment. As expected, treatment with 5-NIdR had no effect on cell cycle progression since the analog is essentially inert without the addition of an exogenous DNA damaging agent. However, treatment with temozolomide significantly effects cell cycle progression by causing a significant block at G_2_/M phase without much effect on cells progressing through S-phase. The inability of temozolomide to block progression through S-phase suggests that DNA lesions produced by temozolomide are likely replicated by specialized DNA polymerases such as pol η and/or pol ι during chromosomal replication. Consistent with this model, our studies showed that combining 5-NIdR with temozolomide causes a significant block at S-phase which was attributed to the inhibition of TLS activity. This inhibition causes GBM cells to undergo cell death by mitotic catastrophe rather than through classic apoptotic pathways.

### Animal studies testing the efficacy and safety of nucleobase-modified nucleosides

Pre-clinical animal studies using a xenograft mouse model of GBM were next performed to verify that 5-NIdR effectively increases the therapeutic efficacy of temozolomide with causing overt side effects (Choi et al., 2018b). In these studies, mice were treated with temozolomide (40 mg/kg) alone or combined with 5-NIdR (100 mg/kg) for 5 consecutive days after tumors reached a volume of ∼500 mm^3^. In general, treatment with temozolomide alone had a minimal effect on tumor growth and this was evident by poor animal survival in which 50% of mice treated with temozolomide alone died within 45 days. In contrast, combining 5-NIdR with temozolomide produced significant anti-tumor effects as nearly 70% of mice receiving this combination survived longer than 250 days post-treatment. This was evident as the majority of mice receiving 5-NIdR and temozolomide showed complete tumor regression within 30 days after treatment. Finally, toxicology studies showed that repeat dosing of mice with 500 mg/kg of 5-NIdR *via* intravenous injection produced no adverse hematological effects. In addition, no adverse effects on major organs including brain, heart, liver, and kidney were observed in either male and female mice.

## Conclusion and future directions

This article provides an opinion review for a number of seminal achievements in developing and applying nucleobase-modified nucleotides, analogs that lack conventional hydrogen-bonding interactions associated with Watson-Crick base pair, in various biochemical fields. These analogs were initially used as mechanistic probes to further elucidate how biophysical features such as pi-stacking, shape, size, and nucleobase desolvation regulate the activity and fidelity of DNA polymerases. Results obtained from numerous labs have demonstrated that hydrogen-bonding interactions are not essential for DNA polymerization to occur. However, rather than providing a universal mechanism for polymerization efficiency, results from several laboratories indicate that most DNA polymerases utilize these features differentially during DNA synthesis. This is particular evident during the replication of damaged DNA in which some nucleobase-modified nucleotides are used more effectively compared to their natural counterparts. Equally important, these differences have been used to develop nucleobase-modified nucleosides as novel therapeutic agents against hyperproliferative diseases such as cancer. In the wake of the global COVID-19 pandemic, it will be of great interest to determine if nucleobase-modified nucleosides can be used as effective anti-viral agents. Likewise, it will be interesting to test the efficacy of these nucleoside analogs as potential anti-microbial agents.

Perhaps the most fertile ground for future applications of nucleobase-modified nucleotides is in the area of synthetic biology. Tremendous strides have been made toward expanding the genetic code with the ultimate goal of producing novel proteins containing unnatural amino acids. In addition, nucleobase-modified nucleotides have been imbedded within aptamers to expand their ability to bind diverse molecules (protein, nucleic acids, metabolites, *etc.*) with the goal to develop more effective therapeutic and diagnostic agents. Other future efforts include using nucleobase-modified nucleobases in CrispR systems to improve genetic engineering capabilities. These approaches may be similar to established work using nucleic acids containing nucleobase-modified nucleobases in PCR sequencing efforts. Another area of interest is to integrate nucleobase-modified nucleobases in the development of mRNA-based vaccine development. mRNA vaccines consist primarily of RNA plus water, salt, sugar, and fat imbedded within a lipid nanoparticle to encapsulate the mRNA and assist its efficient delivery into a cell. However, an important feature of vaccine mRNA is the inclusion modified nucleobase designated N1-methylpseudouridine (Karikó et al., 2005; Nance and Meier, 2021). The modified nucleobase is found naturally and has the same hydrogen-bonding potential as uridine. Thus, its presence does not alter the fidelity of protein synthesis by adversely influencing proper interactions with the ribosome during translation. Instead, this modified nucleobase enhances immune evasion and protein production through three interrelated process. First, the modified nucleobase helps reduce the synthesis of “antisense” RNA that can be generated during transcription (Mu et al., 2018). In particular, RNA polymerases can sometimes use mRNA to “self-prime” additional RNA synthesis. This self-priming activity can generate small amounts of duplex “antisense” mRNA that can produce an undesirable immune response. Secondly, the presence of N1-methylpseudouridine in mRNA can impede the formation of secondary structures such as hairpins that can be recognized by immune receptors such as TLR3 and RIG-I (Karikó et al., 2004). Finally, the altered structure and hydrogen bonding interaction by N1-methylpseudouridine can disrupt interactions with immune sensors such as TLR7 with single-stranded segments of synthetic mRNA (Nelson et al., 2020). As our understanding of mRNA vaccines continues to grow, it will be interesting to investigate if synthetic nucleobase-modified analogs can produce better activities. For example, combining *in vitro* translation capabilities of synthetic RNA containing nucleobase-modified nucleobases could produce novel monoclonal antibodies with improved pharmacodynamic and pharmacokinetic properties. These represent just a few examples that could be used in several biomedically-relevant areas. Truly the sky is the limit for nucleobase-modified nucleotides!

## Glossary

A: adenine
C: cytosine
G: guanine
T: thymine
Vmax: maximal velocity
dQTP: 9-methylimidazo[(4,5)-b]pyrimidine
TLS: translesion DNA synthesis
4-MePoTP: 4-methylpyrimid-2-one triphosphate
Ind-TP: indolyl-2′-deoxyribonucleoside triphosphate
5-AITP: 5-aminoindolyl-2′-deoxyribonucleoside triphosphate
5-CITP: 5-carboxyindolyl-2′-deoxyribonucleoside triphosphate
5-FITP: 5-fluoro-indolyl-2′-deoxyribonucleoside triphosphate
5-EtITP: 5-ethyl-indolyl-2′-deoxyribonucleoside triphosphate
5-Ey-ITP: 5-ethylene-indolyl-2′-deoxyribonucleoside triphosphate
5-MeCITP: 5-methylcarboxylindolyl-2′-deoxyribonucleoside triphosphate
5-NITP: 5-nitroindolyl-2′-deoxyribonucleoside triphosphate
5-CHITP: 5-cyclohexylindolyl-2′-deoxyribonucleoside triphosphate
5-CEITP: 5-cyclohexeneindolyl-2′-deoxyribonucleoside triphosphate
5-PhITP: 5-phenylindolyl-2′-deoxyribonucleoside triphosphate
kpol: polymerization rate constant
5-NI: 5-nitroindole
O6MeG: O6-methylguanine
O6BnG: O6-benzylguanine
O6CMG: O6-carboxymethyl guanine
BIMTP: benzimidazole-2′-deoxyribonucleoside triphosphate
BenziTP: benzimidazole-2′-deoxyribonucleoside triphosphate
PCR: polymerase chain reaction
Ds: 7-(2-thienyl)-imidazo [4,5-b]pyridine
Pa: pyrrole-2-carbaldehyde
Px: 2-nitro-4-propynylpyrrole
SELEX: systematic evolution of ligands by exponential enrichment
ExSELEX: Expansion for SELEX
VEGF: vascular endothelial growth factor
IFNγ, interferon-γ
CSR: compartmentalized self-replication
5-NIdR: 5-nitroindolyl-2′-deoxyribonucleoside
5NIC: 3-carboxamide-5-nitroindole
GBM: glioblastoma multiforme
